# The Voluntary Hospitals' Budget

**Published:** 1918-06-22

**Authors:** 


### THE VOLUNTARY HOSPITALS' BUDGET.

In these anxious times our hospitals and kindred institutions must not be allowed to suffer any degree of want that may cripple their efficiency. The health of every man, woman, and child is as precious to the nation as to the individual, and an organisation such as the voluntary system which does so much to secure a high level of health to the community, both by administering directly to the patients and by training the doctors and nurses who look after those who do not use the hospitals, must be maintained. To support these institutions now is an act of charity, and at the same time an act of patriotism of a most useful kind. The hospitals appeal especially to us at the present time when we know what they are doing for our brave wounded sailors and soldiers, to whom particularly those of us who are unable to fight feel such a debt of gratitude ; and while they are, in most cases by means of extra bed accommodation, performing this duty, the utmost is also done for the sick and suffering poor. And all this work is carried out under great difficulty; staffs of doctors, nurses, and all ranks are depleted, those who are left to work do so, in most cases, during much longer hours and at greater pressure than in normal times, while the expensive nature of special supplies, such as drugs, dressings, and instruments, in conjunction with the increased cost of food and daily necessaries, is a heavy drain upon the institutions.

Before the war the ordinary expenditure of the Metropolitan Voluntary Hospitals and Dispensaries usually increases by about £40,000 a year. In 1912 it was about £1,255,000, in 1913 about £1,295,000, and in 1914 about £1,334,000, but in 1915 it rose to £1,485,000, and since it has been rising appreciably. To meet this heavy expenditure the hospitals and dispensaries have to rely upon three main sources of revenue.

First and most important of these are the gifts of the charitable, for which we are now appealing, such as subscriptions, donations, entertainments, grants raised by King Edward's Hospital Fund and the Hospital Sunday and Saturday Funds, and other receipts of a voluntary character. This source of revenue, together with the payments made by patients (including War Office grants for the maintenance of sailors and soldiers), supplies about two-thirds of the amount required for the upkeep of the institutions, and may be taken as worth about £1,100,000 a year. But to reach that figure a renewal of the generosity of friends in the past is necessary, for new friends but take the place of those who are lost by death or changed circumstances. Expenses are increasing by leaps and bounds, and it is improbable that they will be less than £1,800,000, and the £1,100,000 above mentioned would bring in under 12s. 6d. of every £1 required. Secondly, there is the income from investments and property. The value of this is increasing by about £10,000 to £15,000 a year, and should continue to do so. The amount received in 1915 was £368,000, and in 1916 £382,000, so that the figures for 1917 should be about £400,000, which would amount to 4s. 6d. towards each sovereign required.

These two fairly stable sources of income may be expected to provide 17s. in the pound, but the other 3s., or about one-seventh of the total requirements, must be looked for as coming from a more precarious source—indeed, the only remaining one from which revenue can be derived—viz., the gifts left to hospitals in the shape of legacies. These vary in amount from year to year; in a period of ten years they have fallen as low as £240,000, and reached as high as £451,000, the last figure being for 1914. In 1915 the total fell to £242,049 and rose again to £301,282 in 1916. But in any case legacies should be regarded as extraordinary income, and should be utilised to assist in meeting the expenditure required for rebuilding, for improvements requisite to keep pace with the rapid march of science, or for extension where overcrowding has become apparent, all of which items of extraordinary expenditure have now to be met by special appeals to those who already have given largely.

But extraordinary expenditure finds no place in the present budget. Ordinarily about a quarter of a million a year is spent in this way, but all schemes of rebuilding, enlargement, or alterations are postponed until peace-time.

In conclusion, having shown that such support is necessary, may we ask the old supporters of the Metropolitan Hospitals and Dispensaries to give this year, as nearly as possible, as liberally as they have done in the past? And we call upon those who have not hitherto given to remember what the voluntary hospitals have done, and are doing for the outside community, to recognise under what difficulties they are working at present, and to become welcome friends and supporters at a time when it is possible that these charities may have to suffer in consequence of the heavy calls being made in so many other ways upon the purse of the public.

### INCOME.

TABLE SHOWING INCOME OF THE METROPOLITAN HOSPITALS AND DISPENSARIES IN THE TEN YEARS 1907 TO 1916.

| Year | From the Living | | From the Dead | | | | Total Income |
|---|---|---|---|---|---|---|---|
| | Subscriptions, Donations, Patients' Payments, etc. | Percentage of Total | Investments | Percentage of Total | Legacies | Percentage of Total | |
| | £ | | | | £ | | £ |
| 1907 | 653,320 | 48 | 296,218 | 22 | 407,918 | 30 | 1,357,456 |
| 1908 | 712.461* | 56 | 290,491 | 23 | 265,493 | 21 | 1,268,445 |
| 1909 | 701,458 | 57 | 294,206 | 24 | 240.523 | 19 | 1,236,187 |
| 1910 | 703,841 | 50 | 309,320 | 22 | 394,478 | 28 | 1,407,639 |
| 1911 | 738,622 | 53 | 329,253 | 24 | 322.949 | 23 | 1,390,824 |
| 1912 | 741,439 | 55 | 338,425 | 25 | 272,090 | 20 | 1,351,954 |
| 1913 | 845,908* | 52 | 349,295 | 22 | 424,339 | 26 | 1,619,542 |
| 1914 | 801.239 | 49 | 366,841 | 23 | 451,569 | 28 | 1,619,649 |
| 1915 | 978,294 | 62 | 368,536 | 23 | 242.049 | 15 | 1,588,879 |
| 1916 | 1,143,417 | 63 | 382,377 | 21 | 301,282 | 16 | 1,827,076 |

* Includes £72,565 and £103,970, the amounts received by the London Hospital in 1908 and 1913 respectively as the result of its Quinquennial Appeals in those years.

